# The World Health Organization’s Health Promoting Schools framework: a Cochrane systematic review and meta-analysis

**DOI:** 10.1186/s12889-015-1360-y

**Published:** 2015-02-12

**Authors:** Rebecca Langford, Christopher Bonell, Hayley Jones, Theodora Pouliou, Simon Murphy, Elizabeth Waters, Kelli Komro, Lisa Gibbs, Daniel Magnus, Rona Campbell

**Affiliations:** 1 grid.5337.20000000419367603School of Social & Community Medicine, University of Bristol, 39 Whatley Rd, Bristol, BS8 2PS UK; 2 grid.83440.3b0000000121901201Social Science Research Unit, Institute of Education, University College London, 20 Bedford Way, London, WC1H 0AL UK; 3 grid.5600.30000000108075670Cardiff School of Social Sciences, Cardiff University, 1-3 Museum Place, Cardiff, CF10 3BD UK; 4 grid.1008.9000000012179088XJack Brockhoff Child Health & Wellbeing Program, Melbourne School of Population and Global Health, University of Melbourne, 207 Bouverie Street, Carlton 3053 Melbourne, Australia; 5 grid.15276.370000000419368091Health Outcomes and Policy, Institute for Child Health Policy, University of Florida, 1329 SW 16th Street, Gainesville, FL 32610-0177 USA

**Keywords:** Child health, Adolescent health, Public health, Health promotion, Schools, Interventions, Systematic review

## Abstract

**Background:**

Healthy children achieve better educational outcomes which, in turn, are associated with improved health later in life. The World Health Organization’s Health Promoting Schools (HPS) framework is a holistic approach to promoting health and educational attainment in school. The effectiveness of this approach has not yet been rigorously reviewed.

**Methods:**

We searched 20 health, education and social science databases, and trials registries and relevant websites in 2011 and 2013.

We included cluster randomised controlled trials. Participants were children and young people aged four to 18 years attending schools/colleges. HPS interventions had to include the following three elements: input into the curriculum; changes to the school’s ethos or environment; and engagement with families and/or local communities.

Two reviewers identified relevant trials, extracted data and assessed risk of bias. We grouped studies according to the health topic(s) targeted. Where data permitted, we performed random-effects meta-analyses.

**Results:**

We identified 67 eligible trials tackling a range of health issues. Few studies included any academic/attendance outcomes. We found positive average intervention effects for: body mass index (BMI), physical activity, physical fitness, fruit and vegetable intake, tobacco use, and being bullied. Intervention effects were generally small. On average across studies, we found little evidence of effectiveness for zBMI (BMI, standardized for age and gender), and no evidence for fat intake, alcohol use, drug use, mental health, violence and bullying others. It was not possible to meta-analyse data on other health outcomes due to lack of data. Methodological limitations were identified including reliance on self-reported data, lack of long-term follow-up, and high attrition rates.

**Conclusion:**

This Cochrane review has found the WHO HPS framework is effective at improving some aspects of student health. The effects are small but potentially important at a population level.

## Background

This article is based on a Cochrane Review published in the *Cochrane Database of Systematic Reviews* (*CDSR*) 2014, Issue 4, DOI:10.1002/14651858.CD008958.pub2 (see www.thecochranelibrary.com for information). Cochrane Reviews are regularly updated as new evidence emerges and in response to feedback, and the *CDSR* should be consulted for the most recent version of the review.

Childhood and adolescence are profoundly important for public health. These years are key periods of biological and social change, laying the foundations for future adult health and economic well-being. The influence of childhood experiences on health later in life is well documented [1-6], with attitudes and behaviours acquired then ‘tracking’ into adulthood [7-9]. Establishing positive early childhood experiences in health and education has been highlighted by the World Health Organization (WHO) as key to reducing global health inequities [10]. As noted by Sawyer [11:1631] in a special issue of *The Lancet* on adolescent health, ‘many opportunities for prevention of non-communicable diseases, mental disorders, and injuries in adults arise from a focus on risk processes that begin in or before adolescence’. Promoting health during this early period of life is key to many public health agendas [11].

Given the significance of this period of the life course, schools are an important setting for health promotion, offering a comprehensive, sustained and efficient means of reaching this population. Because almost all children obtain some years of schooling, health promotion in schools can help reduce health inequities. Having a healthy, happy student body is also important for learning: healthy children achieve better educational outcomes which, in turn, are associated with improved health later in life [12].

The WHO’s Health Promoting Schools (HPS) framework, developed in the late 1980s, is underpinned by this reciprocal relationship between health and education. It seeks to overcome the limited success of traditional ‘health education’, establishing instead a holistic approach to promoting health in schools. Although definitions vary, the three key characteristics of a Health Promoting School are set out in Table 1.

**Table 1:** **The Health Promoting Schools framework**

***School curriculum***	Health education topics are promoted through the formal school curriculum.
***Ethos and/or environment***	Health and well-being of students are promoted through the ‘hidden’ or ‘informal’ curriculum, which encompasses the values and attitudes promoted within the school and the physical environment and setting of the school.
***Families and/or communities***	Schools seek to engage with families, outside agencies and the wider community in recognition of the importance of these other spheres of influence on children’s health.

This approach has proved popular and has been implemented in numerous countries worldwide in the absence of clear evidence of its effectiveness or potential harms. A systematic review [13] conducted in 1999 suggested there were ‘limited but promising’ data that this approach could benefit student health. However, the conclusions of the review were limited by the small number of studies available, methodological weaknesses in the trials, and inclusion of non-randomised studies.

Focusing on studies with rigorous experimental evaluation designs, we sought to re-assess the current evidence of effectiveness of the HPS framework for improving the health and well-being of students and their academic achievement.

## Methods

This paper is an abridged version of the associated Cochrane systematic review [14], where full details of the methods and results can be found. A protocol [15] for this review was published in The Cochrane Library and reporting of it adheres to PRISMA [16] guidelines.

### Inclusion criteria

We included cluster randomised controlled trials (RCTs), with clusters at the level of school, district or other geographical area. Participants were students aged 4–18 years attending schools/colleges. To be eligible, interventions had to demonstrate active engagement in all three HPS domains listed in Table 1. Control schools offered no intervention or standard practice, or implemented an alternative intervention including only one or two of the HPS criteria. Primary and secondary outcomes of the review are described in Table 2.

**Table 2:** **Review outcomes**

***Primary health outcomes***	Overweight/obesity; physical activity and sedentary behaviours; nutrition; tobacco use; alcohol use; substance use; sexual health; mental health; violence; bullying; infectious disease (e.g. diarrhoea, respiratory infections); safety and accident prevention; body image/eating disorders; sun safety; and oral health.
***Primary educational outcomes***	Academic achievement, including student standardised test scores or school-level academic achievement.
***Secondary outcomes included***	School attendance outcomes; other school-related outcomes (such as school climate or attachment to school).

### Search strategy

We searched the following databases and trials registries using broad and inclusive search terms to identify all eligible studies: ASSIA, Australian Education Index, British Education Index, BiblioMap, CAB Abstracts, Campbell Library, CENTRAL, CINAHL, Database of Educational Research, EMBASE, Education Resources Information Centre, Global Health Database, International Bibliography of Social Sciences, Index to Theses in Great Britain and Ireland, MEDLINE, PsycINFO, System for Information on Grey Literature in Europe, Social Science Citation Index, Sociological abstracts, TRoPHI, clinicaltrials.gov, Current Controlled Trials, and International Clinical Trials Registry Platform. We also searched relevant websites and reference lists of relevant articles. Searches were conducted in 2011 and 2013. No date or language restrictions were applied.

One author performed an initial title screen, with a second author screening a randomly-selected 10% of titles for quality assurance purposes (kappa score = 0.88). Thereafter, two reviewers independently screened abstracts and full texts to determine eligibility.

### Data extraction

For each study, two authors independently extracted data on participant and intervention characteristics and outcome measures. Where data were missing, we contacted study investigators.

We assessed risk of bias using the Cochrane tool [17]. Two authors independently assessed each study for potential selection, performance, detection, attrition, reporting or other biases.

For analysis, we grouped HPS interventions according to the health topic(s) targeted. For example, we distinguished between studies that sought to tackle obesity by targeting physical activity, those targeting nutrition and those targeting both together. We also identified multiple risk behaviour interventions which targeted multiple health outcomes with one intervention.

### Statistical analysis

For dichotomous data, we used odds ratios to summarise study results. Continuous outcomes were summarised using a mean difference or standardised mean difference (SMD) when outcomes were reported on different scales. Where studies had not adjusted for clustering, we obtained intra-cluster correlation coefficients (ICCs) and mean cluster size from study investigators, allowing us to adjust standard errors for clustering prior to incorporation in meta-analyses. If unavailable, standard deviations (SDs) and ICCs were imputed based on similar studies.

Results were pooled within outcome and intervention types using random-effects meta-analyses where sufficient data were available. We quantified heterogeneity using τ (the between-study SD in effect sizes) and I^2^ [18]. Subgroup and sensitivity analyses were performed, as described in the full report [14].

## Results

**Figure 1:**
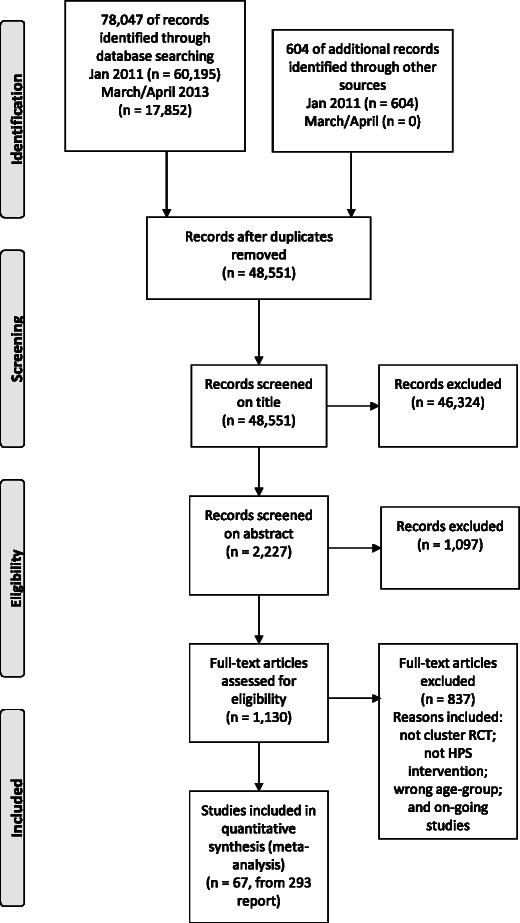
**Flow chart of study selection process.**

Searches yielded 48 551 records, from which we identified 67 eligible studies (Figure 1). Summary details of the types of interventions are presented in Table 3.

**Table 3:** **Characteristics of the trials included in the review, by intervention focus**

**Authors**	**Name**	**Review outcomes**	**Country**	**Target group**	**Duration for study design**	**Theory**
**Nutrition Interventions**						
Anderson 2005 [19]	-	Nutrition	UK	6-7 and 10–11 year- olds	8 months	Health Promoting Schools framework
Bere 2006 [20]	Fruits and Vegetables Make the Mark	Nutrition	Norway	Grade 6	6 months	Social cognitive theory
Evans 2013 [21]	Project Tomato	Nutrition	UK	Year 2	10 months	Framework for health maintenance behaviour
Foster 2008 [22]	School Nutrition Policy Initiative	Obesity/overweight, Nutrition	USA	Grades 4-6	2 years	None stated
Hoffman 2010 [23]	Athletes in Service, Fruit and Vegetable Promotion Program	Nutrition	USA	Kindergarten and Grade 1	2.5 years	Social learning theory
Hoppu 2010 [24]	-	Nutrition	Finland	Grade 8	8 months	Social cognitive theory
Lytle 2004 [25]	TEENS	Nutrition	USA	Grades 7-8	2 years	Social cognitive theory
Nicklas 1998 [26]	Gimme 5	Nutrition	USA	Grade 9	3 years	PRECEDE Model of Health Education
Perry 1998 [27]	5 A DAY Power Plus	Nutrition	USA	Grades 4-5	6 months	Social learning theory
Radcliffe 2005 [28]	-	Nutrition	Australia	Grade 7	11 months	Health Promoting Schools framework
Reynolds 2000 [29]	High 5	Nutrition	USA	Grade 4	1 year	Social cognitive theory
Te Velde 2008 [30]	Pro Children Study	Nutrition	Netherlands, Norway, Spain	Grades 5-6	2 years	Social cognitive theory, Ecological model
**Physical Activity Interventions**						
Eather 2013 [31]	Fit-4-Fun	Obesity/overweight, Physical Activity	Australia	Grades 5-6	8 weeks	Health Promoting Schools framework, Social cognitive Theory, Harter’s Competence Motivation Theory
Kriemler 2010 [32]	KISS	Obesity/overweight, Physical Activity	Switzerland	Grades 1 and 5	11 months	None stated
Simon 2006 [33]	ICAPS	Obesity/overweight, Physical Activity	France	Grade 6	4 years	“Theory based” but no details of a named theory provided
Wen 2008 [34]	-	Physical Activity	Australia	Years 4-5	2 years	Health Promoting Schools framework
**Physical Activity + Nutrition Interventions**						
Arbeit 1992 [35]	Heart Smart	Obesity/overweight, Physical Activity, Nutrition	USA	Grades 4-5	2.5 years	Social cognitive theory
Brandstetter 2012 [36]	URMEL ICE	Obesity/overweight, Physical Activity, Nutrition	Germany	Grade 2	9 months	Social cognitive theory
Caballero 2003 [37]	Pathways	Physical Activity, Nutrition	USA	Grade 3	3 years	Social learning theory
Colín-Ramírez 2010 [38]	RESCATE	Obesity/overweight, Physical Activity, Nutrition	Mexico	Grades 4-5	1 year	None stated
Crespo 2012 [39]	Aventuras para Niños	Obesity/overweight, Physical Activity, Nutrition	USA	Kindergarten-Grade 2	5 semesters	Social ecological theory, Social cognitive theory, Health belief model, structural model of health behavior
Foster 2010 [40]	HEALTHY	Obesity/overweight	USA	Grades 6-8	3 years	None stated
Grydeland 2013 [41]	Health in Adolescents (HEIA)	Obesity/overweight, Physical Activity, Nutrition	Norway	Grade 6	20 months	Socio-ecological framework
Haerens 2006 [42]	-	Obesity/overweight, Physical Activity	Belgium	Grades 7-8	2 years	Theory of planned behaviour, Transtheoretical model, Social cognitive theory, Attitude, social influence and self-efficacy (ASE) model
Jansen 2011 [43]	Lekker Fit	Obesity/overweight, Physical Activity	Netherlands	Grades 3-8	8 months	Theory of planned behaviour, Ecological model
Llargues 2011 [44]	AVall	Obesity/overweight, Physical Activity, Nutrition	Spain	5-6 year olds	2 years	Educational methodology ‘IVAC’
Luepker 1996 [45]	CATCH	Physical Activity, Nutrition	USA	Grade 3	3 years	Social cognitive theory, Social learning theory
Rush 2012 [46]	Project Energize	Obesity/overweight	New Zealand	5 and 10 year olds	2 years	Health Promoting Schools framework
Sahota 2001 [47]	APPLES	Obesity/overweight, Physical Activity, Nutrition	UK	Years 4-5	10 months	Health Promoting Schools framework
Sallis 2003 [48]	M-SPAN	Physical Acivity, Nutrition	USA	Grades 6-8	2 years	Ecological model
Shamah Levy 2012 [49]	Nutrición en Movimiento	Obesity/overweight, Nutrition	Mexico	Grade 5	6 months	Not explicitly theory-based, but mentions use of theory of peer learning for one element of the intervention (puppet theatre)
Trevino 2004 [50]	Bienestar (1)	Physical Activity, Nutrition	USA	Grade 4	5 months	Social cognitive theory, Social ecological theory
Trevino 2005 [51]	Bienestar (2)	Obesity/overweight, Physical Activity	USA	Grade 4	8 months	Social cognitive theory
Williamson 2012 [52]	Louisiana (LA) HEALTH	Obesity/overweight, Physical Activity, Nutrition	USA	Grades 4-6	2.5 years	Social learning theory
**Tobacco Interventions**						
De Vries (Denmark) 2003 [53]	ESFA (Denmark)	Tobacco	Denmark	Grade 7	3 years	Attitude, social influence and self-efficacy (ASE) model
De Vries (Finland) 2003 [53]	ESFA (Finland)	Tobacco	Finland	Grade 7	3 years	Attitude, social influence and self-efficacy (ASE) model
Hamilton 2005 [54]	-	Tobacco	Australia	Grade 9	2 years	Health Promoting Schools framework
Perry 2009 [55]	Project MYTRI	Tobacco	India	Grades 6-8	2 years	Social cognitive theory, social influences model
Wen 2010 [56]	-	Tobacco	China	Grades 7-8	2 years	Socio-ecological framework, PRECEDE-PROCEED model
**Alcohol Interventions**						
Komro 2008 [57]	Project Northland (Chicago)	Alcohol, Tobacco, Drugs	USA	Grade 6-8	3 years	Theory of triadic influence
Perry 1996 [58]	Project Northland (Minnesota)	Alcohol, Tobacco, Drugs	USA	Grades 6-8	3 years	Social learning theory
**Multiple Risk Behaviour Interventions**						
Beets 2009 [59]	Positive Action (Hawai’i)	Tobacco, alcohol, drugs, violence, sexual health, academic and school-related outcomes	USA	Grades 2-3	3 years	Theory of self-concept, Theory of triadic influence
Eddy 2003 [60]	LIFT	Tobacco, alcohol, drugs	USA	Grades 1 and 5	10 weeks	Coercion theory
Flay 2004 [61]	Aban Aya	Violence, drugs, sexual health	USA	Grade 5	4 years	Theory of triadic influence
Li 2011 [62]	Positive Action (Chicago)	Tobacco, alcohol, drugs, violence, academic and school-related outcomes	USA	Grade 3	6 years	Theory of self-concept, Theory of triadic influence
Perry 2003 [63]	DARE Plus	Tobacco, alcohol, drugs, violence	USA	Grade 7	2 years	Theory of triadic influence
Schofield 2003 [64]	Hunter Region Health Promoting Schools Program	Tobacco	Australia	Years 7-8	2 years	Health Promoting Schools framework Community Organization Theory
Simons-Morton 2005 [65]	Going Places	Tobacco, alcohol	USA	Grades 6-8	3 years	Social cognitive theory
**Sexual health Interventions**						
Basen-Engquist 2001 [66]	Safer Choices	Sexual health	USA	Grade 9	2 years	Social cognitive theory, social influence theory and models of school change
Ross 2007 [67]	MEMA Kwa Vijana	Sexual health	Tanzania	Students aged 14+ years	3 years	Social learning theory
**Mental Health and Emotional Well-being Interventions**						
Bond 2004 [68]	Gatehouse Project	Mental health and emotional well-being, tobacco, drugs, bullying	Australia	Grade 8	3 years	Health Promoting schools framework, attachment theory
Sawyer 2010 [69]	beyondblue	Mental health and emotional well-being	Australia	Year 8	3 years	Health Promoting Schools framework
**Violence Prevention Interventions**						
Orpinas 2000 [70]	Students for Peace	Violence	USA	Grades 6-8	3 semesters	Social cognitive theory
Wolfe 2009 [71]	Fourth R	Violence, sexual health	Canada	Grade 9	15 weeks	None stated
**Anti-bullying Interventions**						
Cross 2011 [72]	Friendly Schools	Bullying	Australia	Grade 4	2 years	Health Promoting Schools framework, Social cognitive theory, Ecological theory, Social control theory, Health belief model, Problem behaviour theory
Cross 2012 [73]	Friendly Schools, Friendly Families	Bullying	Australia	Grades 2, 4 and 6	2 years	Health Promoting Schools framework
Fekkes 2006 [74]	-	Bullying	Netherlands	9-12 year-olds	2 years	No specific theory but based on Olweus bullying programme
Frey 2005 [75]	Steps to Respect	Bullying	USA	Grades 3-6	1 year	None stated
Kärnä 2011 [76]	KiVa (1)	Bullying	Finland	Grade 4-6	9 months	Social cognitive theory
Kärnä 2013 [77]	KiVa (2)	Bullying	Finland	Grade 1–3 and 7-9	9 months	Social cognitive theory
Stevens 2000 [78]	-	Bullying	Belgium	10 to 16 year-olds	Not clear	Social learning theory
**Hand-washing Interventions**						
Bowen 2007 [79]	-	Illness from infectious diseases	China	Grade 1	5 months	None stated
Talaat 2011 [80]	-	Illness from infectious diseases	Egypt	Grades 1–3 (for data collection, but all children in school targeted)	12 weeks	None stated
**Miscellaneous Interventions**						
Hall 2004 [81]	School Bicycle Safety Project/The Helmet Files	Safety/accidents	Australia	Grade 5	2 years	Health Promoting Schools framework
McVey 2004 [82]	Healthy Schools- Healthy Kids	Body image	Canada	Grade 6-7	8 months	Health Promoting Schools framework, Ecological approach
Olson 2007 [83]	SunSafe	Sun safety	USA	Grades 6-8	3 years	Social cognitive theory, Socio-ecological theory, Protection motivation theory
Tai 2009 [84]	-	Oral health	China	Grade 1	3 years	Health Promoting Schools framework

BMI = Body Mass Index; zBMI = Body Mass Index, standardised for age and gender.

Twenty-nine studies were conducted in North America (27 USA [22,23,25-27,29,35,37,39,40,45,48,50-52,57-63,65,66,70,75,83], 2 in Canada [71,82]), 19 in Europe [19-21,24,30,32,33,36,41,42,44,47,53,74,76-78,85], 11 in Australasia [28,31,34,46,54,64,68,69,72,73,81] and eight in middle- or low-income countries (China [56,79,84], India [55], Mexico [38,49], Egypt [80] and Tanzania [67]). Thirty-four studies focused on physical activity and/or nutrition. Seven focused on bullying, five on tobacco and two each targeted alcohol, mental health, violence, sexual health, and hand-hygiene. Seven studies evaluated multiple risk behaviour interventions. The remaining four studies focused on accident prevention, eating disorders, sun protection and oral health.

The quality of evidence was variable, both between studies and across the different domains of potential bias [14]. Poor reporting hampered our ability to assess risk of bias, particularly regarding random sequence generation, where the majority of studies were assessed as being at unclear risk of bias. Because in most studies all clusters were randomly allocated simultaneously, we deemed these at low risk of allocation concealment bias. It was difficult to blind participants to the fact they were participating in an intervention. As the majority of outcomes were measured by self-report, these were deemed to be at high risk of performance and detection bias due to lack of blinding. Where objective measures were reported (e.g. BMI), eight studies reported assessors were blind to group allocation. We assessed 34 studies as being at high risk of attrition bias. Lack of published protocols hampered our ability to assess risk of selective reporting of outcome data. Twenty-nine studies were rated as at high risk of other bias, largely relating to the external validity of the trials.

### Impact on health outcomes

Table 4 presents summary effect estimates from meta-analyses for each health outcome. Details of the effect for each study, forest plots for each analysis and subgroup and sensitivity analyses are presented in full in the Cochrane review [14].

**Table 4:** **Summary estimates (95% CIs) for health outcomes meta-analyses**

**Outcome**	**Intervention focus**	**Number of studies**	**Intervention participants**	**Control participants**	**Mean difference**	**95% CI**	**I** ^**2**^	**τ**
**BMI**	*Nutrition only*	1	479	364	−0.04	−0.28, 0.20	n/a	n/a
	*Physical Activity only*	3	772	658	−0.38	−0.73, −0.03*	86%	0.08
	*Physical Activity & Nutrition*	9	6520	7108	−0.11	−0.24, 0.02	84%	0.03
**zBMI**	*Nutrition only*	1	479	364	−0.01	−0.09, 0.07	n/a	n/a
	*Physical Activity only*	1	102	94	−0.47	−0.69, −0.25*	n/a	n/a
	*Physical Activity & Nutrition*	7	5672	5512	0	−0.04, 0.03	41%	0
					**Standardised mean diff.**	**95% CI**	**I** ^**2**^	**Tau**
**Physical Activity**	*Nutrition only*	1	416	335	0.02	−0.02, 0.06	n/a	n/a
	*Physical Activity only*	2	671	563	0.17	−0.16, 0.50	93%	0.05
	*Physical Activity & Nutrition*	6	3244	2946	0.14	0.03, 0.26*	66%	0.01
**Physical Fitness**	*Physical Activity only*	2	396	298	0.35	−0.20, 0.90	95%	0.15
	*Physical Activity & Nutrition*	3	2059	2171	0.12	0.04, 0.20*	0%	0
**Fat intake**	*Nutrition only*	7	2205	2011	−0.08	−0.21, 0.05	68%	0.02
	*Physical Activity & Nutrition*	10	6498	5962	−0.04	−0.20, 0.12	95%	0.06
**Fruit & Vegetable intake**	*Nutrition only*	9	3293	2917	0.15	0.02, 0.29*	83%	0.03
	*Physical Activity & Nutrition*	4	3507	3105	0.04	−0.18, 0.26	79%	0.04
**Depression**	*Emotional well-being*	2	3252	2847	0.06	−0.00, 0.13	0%	0
	*Anti-bullying*	1	1106	1118	0	−0.08, 0.08	n/a	n/a
					**Odds ratio**	**95% CI**	**I** ^**2**^	**Tau**
**Tobacco use**	*Tobacco only*	3	2244	2503	0.77	0.64, 0.93*	16%	0
	*Multiple Risk Behaviour*	5	5503	4489	0.84	0.76, 0.93*	0%	0
	*Emotional well-being*	1	315	315	0.79	0.59, 1.06	n/a	n/a
	*Alcohol only*	1	1005	896	0.74	0.61, 0.90*	n/a	n/a
**Alcohol use**	*Alcohol only*	2	3506	3975	0.72	0.34, 1.52	82%	0.25
	*Multiple Risk Behaviour*	4	4496	3644	0.75	0.55, 1.02	78%	0.07
	*Emotional well-being*	1	809	810	1.13	0.76, 1.67	n/a	n/a
**Substance use**	*Multiple Risk Behaviour*	3	3804	3016	0.57	0.29, 1.14	71%	0.26
	*Alcohol only*	2	3506	3975	0.94	0.78, 1.12	0%	0
	*Emotional well-being*	1	233	233	0.81	0.57, 1.15	n/a	n/a
**Violence**	*Violence prevention*	1	929	1161	1.13	0.61, 2.07	n/a	n/a
	*Multiple Risk Behaviour*	3	3806	3014	0.5	0.23, 1.09	93%	0.42
**Being bullied**	*Anti-bullying*	6	13993	12263	0.83	0.72, 0.96*	61%	0.02
	*Multiple Risk Behaviour*	1	2635	2108	0.97	0.90, 1.05	n/a	n/a
	*Emotional well-being*	1	481	482	0.88	0.68, 1.13	n/a	n/a
**Bullying others**	*Anti-bullying*	6	13949	12227	0.9	0.78, 1.04	67%	0.02
	*Multiple Risk Behaviour*	1	195	168	0.49	0.34, 0.71*	n/a	n/a

*95% confidence intervals do not include the null.

BMI = Body Mass Index; zBMI = Body Mass Index, standardised for age and gender.

### BMI/zBMI

On average, *Physical Activity* interventions were able to reduce BMI in intervention students by 0.38 kg/m^2^ (95% CI 0.03 to 0.73), relative to control schools (Table 4). Although heterogeneity was large (I^2^ = 86%) all three studies gave evidence in favour of the intervention. Nine studies targeted *Physical Activity + Nutrition* and also showed an average reduction in BMI of 0.11 kg/m^2^ but with a wide confidence interval crossing the null (95% CI −0.24 to 0.02). The single nutrition intervention [22] measuring BMI did not show any impact.

When zBMI was used (which accounts for age and gender), only the single *Physical Activity* intervention [31] showed an effect (MD = 0.47, 95% CI −0.69 to −0.25). No effect was found for the *Nutrition-only* or the *Physical Activity + Nutrition* interventions.

### Physical activity/fitness

On average, there was evidence that *Physical Activity + Nutrition* interventions produced a small increase in physical activity in students relative to controls (SMD = 0.14, 95% CI 0.03 to 0.26) but again, heterogeneity was high (I^2^ = 66%) (Table 4). The two *Physical Activity* interventions showed differing results with one favouring the intervention [33] and the other showing no effect [32]. No effect was seen for the single *Nutrition-only* intervention [22].

For physical fitness, there was evidence that *Physical Activity + Nutrition* interventions were effective at increasing fitness levels in students (SMD = 0.12, 95% CI 0.04, 0.20). The two *Physical Activity* interventions [31,32] also showed a positive estimated effect but with a large amount of heterogeneity (I^2^ = 95%) and a wide confidence interval crossing the null (SMD = 0.35, 95% CI −0.20, 0.90).

### Nutrition

High levels of heterogeneity were observed for both fruit and vegetable intake, and fat intake outcomes. *Nutrition-only* interventions were effective on average in increasing reported fruit and vegetable intake among students (SMD = 0.15, 95% CI 0.02 to 0.29, I^2^ = 83%), but not for reducing fat intake. On average, *Physical Activity + Nutrition* interventions had no effect on fat intake or fruit and vegetable intake.

### Tobacco

There was good evidence that both *Tobacco-only* (OR = 0 · 77, 95% CI 0.64 to 0.93, I^2^ = 16%) and *Multiple Risk Behaviour* (OR = 0 · 84, 95% CI 0.76 to 0.93, I^2^ = 0%) interventions are effective in reducing smoking (Table 4). The alcohol intervention [58], which also looked at the impact on tobacco use, also showed a positive intervention effect (OR = 0.74, 95% CI 0.61 to 0.9). The single *Emotional well-being* intervention gave an estimated effect in favour of the intervention (OR = 0.79) but with a wide confidence interval (95% CI 0.59 to 1.06).

### Alcohol

Although some individual studies showed an effect on reducing alcohol intake, on average there was no evidence of an effect (Table 4). The two *Alcohol-only* interventions produced conflicting results, with confidence intervals that do not overlap: one suggesting a positive effect of the intervention on alcohol intake [58] (OR = 0.45, 95% CI 0.24 to 0.87) and the other suggesting no effect [57] (OR = 0.99, 95% CI 0.97 to 1.01). The *Multiple Risk Behaviour* interventions similarly produced conflicting results, with the two *Positive Action* trials both indicating a positive effect (OR = 0.48, 95% CI 0.32 to 0.73 [59]; OR = 0.44, 95% CI 0.21 to 0.92 [62]) but the remaining two studies finding no effect. The *Emotional well-being* intervention similarly found no effect.

### Drugs

Neither the *Alcohol-only* interventions [57,58] nor the *Emotional well-being* intervention [68] showed evidence of effectiveness in reducing substance use (Table 4). One *Multiple Risk Behaviour* intervention [59] found a positive effect on substance use (OR = 0.28, 95% CI 0.13 to 0.63) but the other two studies reported inconclusive results [62,63], so that the estimate of average effect had a wide confidence interval (OR = 0.57, 95% CI 0.29 to 1.14).

### Mental health

There was no evidence that these interventions were effective in reducing depression (Table 4). Indeed, for the two studies focusing specifically on mental health and emotional well-being, estimated effect sizes were in the opposite direction with intervention students reporting slightly poorer mental health (OR = 0.06, 95% CI 0.00 to 0.13). The *Anti-bullying* intervention [74] found no effect on levels of depression in students.

### Violence

There was no evidence that *Violence Prevention* or *Multiple Risk Behaviour* interventions were effective in reducing violent behaviour (Table 4). The *Violence Prevention* intervention [70] found no effect on student violence. The *Multiple Risk Behaviour* interventions produced conflicting results. The two *Positive Action* trials both found evidence of a reduction in violent behaviours (OR = 0.32, 95% CI 0.16 to 0.32 [59]; OR = 0.38, 95% CI 0.25 to 0.56 [62]). However, another much larger study [63] found no effect. The resulting pooled estimate from a random-effects meta-analysis had a very wide confidence interval (OR = 0.50, 95% CI 0.23 to 1.09).

### Bullying

*Anti-bullying* interventions showed an average reduction of 17% for reports of being bullied (OR = 0.83, 95% CI 0.72 to 0.96, I^2^ = 61%) relative to controls, although there was considerable heterogeneity (Table 4). For *bullying others*, the confidence interval for the pooled effect crossed the null (OR = 0.90, 95% CI 0.78 to 1.04, I^2^ = 67%), but the two largest studies [76,77] investigating the same intervention showed strong evidence of an effect. The *Emotional well-being* intervention [68] failed to show impact on both being bullied or bullying others. No effect was seen for being bullied for the single *Multiple Risk Behaviour* intervention [63] reporting this outcome. However, another *Multiple Risk Behaviour* intervention [62] reported the effect of their intervention on bullying others and found evidence of a large reduction (OR = 0.49, 95% CI 0.34 to 0.71).

### Other health outcomes

We were unable to meta-analyse results from studies for the following topics due to paucity of data: sexual health, hand-hygiene, accident prevention, eating disorders, sun safety and oral health. Results from these interventions are summarised in the Cochrane review [14].

### Educational outcomes

Very few studies presented any academic or attendance outcomes. Two studies collected data on standardised scores for reading and maths [59,62]. They also presented data on suspensions and retentions in grades, student disaffection and teachers’ perceptions of student motivation and performance. Only four studies [59,62,79,80] presented student absenteeism data. Educational outcome results are summarised in Table 5.

**Table 5:** **Summary of impact on educational outcomes**

**Study reference**	**Intervention type**	**Authors’ conclusions**
Li 2011 [62]	Multiple Risk Behaviour	Positive intervention effects found for: student disaffection with learning (P < 0.01); teachers’ ratings of academic motivation (P < 0.05); absenteeism rates (P < 0.05)
		No effect found for: teachers’ ratings of academic performance; standardised test scores for reading and maths
Beets 2009 [59]	Multiple Risk Behaviour	Positive intervention effects found for: standardised test scores for reading and maths (P = 0.043 and 0.006, respectively); absenteeism (P = 0.001); suspensions (P = 0.028); student, teacher and parent ‘School Quality Composite’ scores (P = 0.015, 0.006 and 0.007, respectively)
		No effect found for: student retentions in grade
Fekkes 2006 [74]	Anti-bullying	No effect found for: general satisfaction with school life; satisfaction with contact with other pupils; or satisfaction with contact with teachers
Bond 2004 [68]	Mental health	Positive intervention effect found for: school attachment (Adj. OR 1.33, 95%CI 1.02 to 1.75; un-adjusted OR non-significant)
Sahota 2001 [47]	Physical Activity and Nutrition	No effect found for: self-perceived scholastic competence
Sawyer 2010 [69]	Mental health	Positive intervention effect found for: teacher ratings of school climate over time (intervention x time β = 0.43, P < 0.05)
		No effect found for: student rating of school climate
Bowen 2007 [79]	Hand hygiene	Positive intervention effect found for: attendance. Intervention schools (expanded group) experienced 42% fewer absence episodes (P = .03) and 54% fewer days of absence (P = .03) than control schools
Talaat 2011 [80]	Hand hygiene	Positive intervention effect found for: attendance. Overall, absences caused by illness were reduced by 21% in intervention schools (5.7 vs. 7.2 median episodes, P < 0.001)
Simons-Morton 2005 [65]	Multiple Risk Behaviour	No effect found for: students’ perceptions of school climate
McVey 2004 [82]	Eating disorders	No effect found for: teachers’ perceptions of school climate
Kärnä 2011 [76]	Anti-bullying	Positive intervention effect found for: well-being at school in intervention students (0.096, P = 0.011)

## Discussion

This is the first systematic review of cluster RCTs to assess the effectiveness of the WHO’s Health Promoting Schools framework in improving health and academic achievement in students. We identified 67 eligible studies focusing on a wide range of health issues.

Our analyses showed modest positive intervention effects on average in reducing BMI, smoking and incidence of being bullied, and increasing physical activity, fitness, and fruit and vegetable intake. We found little or no evidence of effect for HPS interventions assessing zBMI, fat intake, alcohol use, drug use, violence, depression or bullying others. Paucity of data meant we were unable to meta-analyse data for outcomes relating to sexual health, hand-washing, oral health, accident prevention or eating disorders.

HPS interventions focusing on physical activity alone found an average reduction in BMI of 0.38 kg/m^2^ but no effect was found for *Physical Activity + Nutrition* interventions. We found little evidence of effect for zBMI, other than in the single *Physical Activity* intervention [31]. *Physical Activity + Nutrition* interventions were, on average, able to produce increases in physical activity and fitness, equivalent to an additional three minutes of moderate-to-vigorous activity per day and a 0.25 level increase in the shuttle run fitness test. Those interventions focused solely on nutrition increased fruit and vegetable intake by an average of 30 g/day or roughly half a portion. These effect sizes are small but are comparable to findings from other school based interventions [86-88]. Small effects scaled up to population level can produce public health benefits [89], although at present these potential gains appear modest. Stronger evidence is available for HPS effects on smoking and bullying. Students receiving HPS interventions were, on average, 23% less likely to smoke and 17% less likely to be bullied.

We found little or no evidence of effect for alcohol use, drug use, violence, depression and bullying others but this should not be confused with ‘evidence of no effect’. For the majority of outcomes, data were available from few studies. Although the confidence intervals for the pooled effect cross the null, for all but one outcome (depression), summary estimates from meta-analyses consistently favour the HPS intervention. Thus, while we cannot at this stage conclude that the HPS approach is effective for these outcomes, there is sufficient evidence of promise to warrant further trials in these areas. Indeed, interventions focusing specifically on school environments have shown promising effects in reducing alcohol use and violence in students [90]. That some individual trials showed evidence of effect for several of these outcomes also suggests the need for exploration into which intervention components might be effective.

The use of cluster RCTs to evaluate complex interventions such as the HPS framework is much debated. Some have argued that RCTs are an inappropriate method to evaluate complex public health programmes, based on an assumption that RCTs require highly standardised intervention components and methods of delivery that preclude the possibility of local adaptation [91,92]. This assumption is unfounded [93,94]. Well-designed cluster RCTS can capture complexity and allow for local adaptation; the critical issue is ‘what’ is standardised (the intervention components or the steps in the change process) [94]. This review of 67 cluster RCTs represents an important contribution to the body of evidence on the effectiveness of the HPS approach. Focusing on the most robust evidence available and using a conservative approach to assess effectiveness, we have found evidence in favour of the HPS framework for a number of important outcomes. To contextualise these findings, it is important this review be read alongside other evaluations of the HPS framework employing different evaluation study designs [95,96] which offer insight into the process and practicalities of implementation.

Our meta-analyses provide the best summary to-date of the likely average effect of HPS interventions. However this review presented methodological challenges. Unlike clinical trials which often involve standardised interventions, homogenous populations and well-established outcome measures, public health interventions display more heterogeneity, particularly in the case of non-prescriptive interventions such as the HPS framework that allows considerable flexibility in intervention components.

Our meta-analyses have limitations. First, where standard deviations (SD) for study populations were not reported, we imputed a SD from another similar study to calculate a standardised mean difference (SMD). Sensitivity analyses examined the impact of this decision on our analyses [14]. Second, to calculate SMDs we used the overall (‘total’) SD across all individuals in a study rather than the ‘within-cluster’ SD, as studies rarely reported the latter. However, because ICCs were found to be small, this is unlikely to have substantially affected our results. Finally, in a minority of studies we used ICCs from similar studies to adjust for clustering, conservatively choosing the largest available ICC.

A further limitation of the review is that we are unable to compare the effectiveness of the HPS approach to simpler, less holistic interventions because most studies compared HPS interventions against no intervention or the school’s usual practice. While HPS effect sizes are broadly similar to results from other school-based systematic reviews [86-88,97,98], the latter often include different types of interventions ranging from ‘curriculum only’ interventions to more comprehensive programmes making meaningful comparisons difficult. Future studies should consider use of factorial designs to identify the relative importance of the three HPS domains and the way in which they interact.

Whilst our review has found evidence in favour of the HPS approach for a number of outcomes, it has also identified gaps in our knowledge base. We lack sufficient data at present to determine the effect of this approach for a number of health outcomes, particularly mental health and sexual health. We also identified an imbalance between which health topics were focused on at different ages. Physical activity and/or nutrition interventions tended to focus on younger children (<12 years) while substance use, violence, sexual health and mental health interventions usually targeted older students (12–14 year-olds). This imbalance is unjustified. Obesity does not just affect younger children [99]; we need to develop effective obesity-prevention interventions for older children too. Equally, two of the most effective *Multiple Risk Behaviour* interventions [59,62] focusing on substance use and violence were conducted in elementary-school children, suggesting that intervening early may help prevent risk-taking in teenage years. We also identified the *family/community* domain to be the weakest aspect of the implementation of the HPS framework with most studies employing very minimal efforts to engage families (e.g. newsletter articles or flyers).

The majority of studies did not provide data on long-term follow-up or economic costs so the sustainability and cost-effectiveness of this approach is largely unknown. Studies were also often underpowered and relied heavily on self-reported data. It is disappointing to note that many of these methodological issues were identified in a previous review of the HPS framework and little improvement has been made in the past fifteen years [13]. Additionally, the current evidence base is predominantly based on studies from high-income contexts, mostly from North America. Only eight studies were conducted outside of high-income countries, and only one took place within a low-income country [67].

The lack of evidence from poorer parts of the world is worrying. Given the well-established links between poor nutrition and infectious disease on children’s cognitive development [100,101], the HPS framework should have much to offer in such contexts. Conversely, the tripling of obesity levels in just twenty years in low- and middle-income countries [102] demands cross-sectoral action, which the HPS framework might help address. Also, aggressive marketing of tobacco in the developing world has led to an increase in smoking, and any substantial increase in adolescent smoking in these contexts will have devastating consequences for future adult health [11:1636]. There are over three billion 0–24 year-olds alive today, almost 90% of whom live in low-and middle-income countries [103]. Investing in child and adolescent health in such contexts is crucial for improving population health and, consequently, national economic development.

Finally, our review highlights the lack of evidence regarding the educational impact of the HPS framework. Only four studies measured academic attainment or student attendance. This is disappointing for two reasons. First, it suggests researchers may have failed to grasp that improving educational outcomes is, in itself, a public health priority. Second, interventions are more likely to be successful and scaled up if educationalists are convinced it will contribute to the core mission of schools: to educate students. The WHO recently highlighted the lack of attention paid to the impact of child health on educational outcomes in high-income countries [12]. Future HPS evaluations should seek to address this gap.

Child and adolescent health matter. Investment in these formative years can prevent suffering, reduce inequity, create healthy and productive adults and deliver social and economic dividends to nations. Schools are an obvious place to facilitate this investment given the inextricable links between health and education [104]. Ultimately the aim of these two disciplines is largely the same: to create healthy, well-educated individuals who can contribute successfully to society.

Despite the obvious connections, across the globe structural barriers prevent the realisation of this mutual agenda. Government departments responsible for health and education often operate in isolation from one another and this fundamental connection is lost. The WHO explicitly set out a new vision of health and education in its HPS framework, yet since its inception there appears to have been little advance in breaking down this siloed approach. Our review demonstrates the potential benefits of this approach for some health outcomes but not others. We have yet to see its benefit for education. This is a political issue. Governments must commit to fostering the meaningful cross departmental working that would allow this policy to achieve its potential.

## Conclusion

This review has found the WHO HPS framework is effective at improving some aspects of student health and shows evidence of promise in improving others. The effects are small but potentially important at a population level.

## Abbreviations

ASSIA: Applied social sciences index and abstracts
BMI: Body mass index
CINAHL: Cumulative index to nursing and allied health literature
HPS: Health promoting schools
ICC: Intra-cluster correlation coefficients
OR: Odds ratio
PRISMA: Preferred reporting items for systematic reviews and meta-analyses
RCT: Randomised controlled trial
SD: Standard deviation
SMD: Standardised mean difference
TRoPHI: Trials register of promoting health interventions
WHO: World health organization
